# Combined pulmonary vein stenosis stenting and left atrial appendage occlusion in a patient with hemoptysis after atrial fibrillation ablation

**DOI:** 10.1186/s12872-020-01483-4

**Published:** 2020-04-22

**Authors:** Yan-Jie Li, Xin Pan, Cheng Wang, Ben He

**Affiliations:** Department of Cardiology, Shanghai Chest Hospital, Shanghai Jiao Tong University, 241 West Huaihai Road, Shanghai, 200030 China

**Keywords:** Atrial fibrillation, Hemoptysis, Left atrial appendage occlusion, Pulmonary vein stenosis, Stenting

## Abstract

**Background:**

Pulmonary vein stenosis (PVS) after radiofrequency ablation for non-valvular atrial fibrillation (AF) is an uncommon but serious complication. PVS stenting can rapidly restore pulmonary flow and improve symptoms with long-term low incidence of restenosis. However, high risk of thrombosis remains if AF is recurrent, especially for CHA_2_DS_2_-VASc > 2.

**Case presentation:**

A 67-year-old man with diabetes, hypertension and a history of stroke underwent radiofrequency pulmonary vein isolation for persistent AF 1 year ago. Six months later he developed recurrent respiratory infection and massive hemoptysis. Computed tomography pulmonary angiography revealed severe left pulmonary vein stenosis. Simultaneous percutaneous PVS stenting and left atrial appendage occlusion were performed to resolve recurrent hemoptysis and prevent stroke. The clinical follow-up indicated a good short and mid-term result with significant improvement of symptoms.

**Conclusions:**

Simultaneous PVS stenting and left atrial appendage occlusion is feasible and effective in patients with recurrence of AF and hemoptysis induced by radiofrequency ablation for AF.

## Background

Pulmonary vein stenosis (PVS) is an uncommon but potentially serious complication following catheter ablation for atrial fibrillation (AF) [1]. Obviously, in symptomatic cases with severe PVS, early PVS stenting is needed to restore pulmonary flow [2, 3]. However, high risk of cardiac thrombosis remains if AF is recurrent. In such situations with co-existing bleeding complication, left atrial appendage occlusion (LAAO), currently regarded as a non-inferior alternative to anticoagulation in patients with non-valvular AF [4, 5], is useful to reduce the risk of thrombosis and bleeding. Here, we report a case of simultaneous percutaneous PVS stenting and LAAO to resolve the dilemma.

## Case presentation

**Fig. 1:**
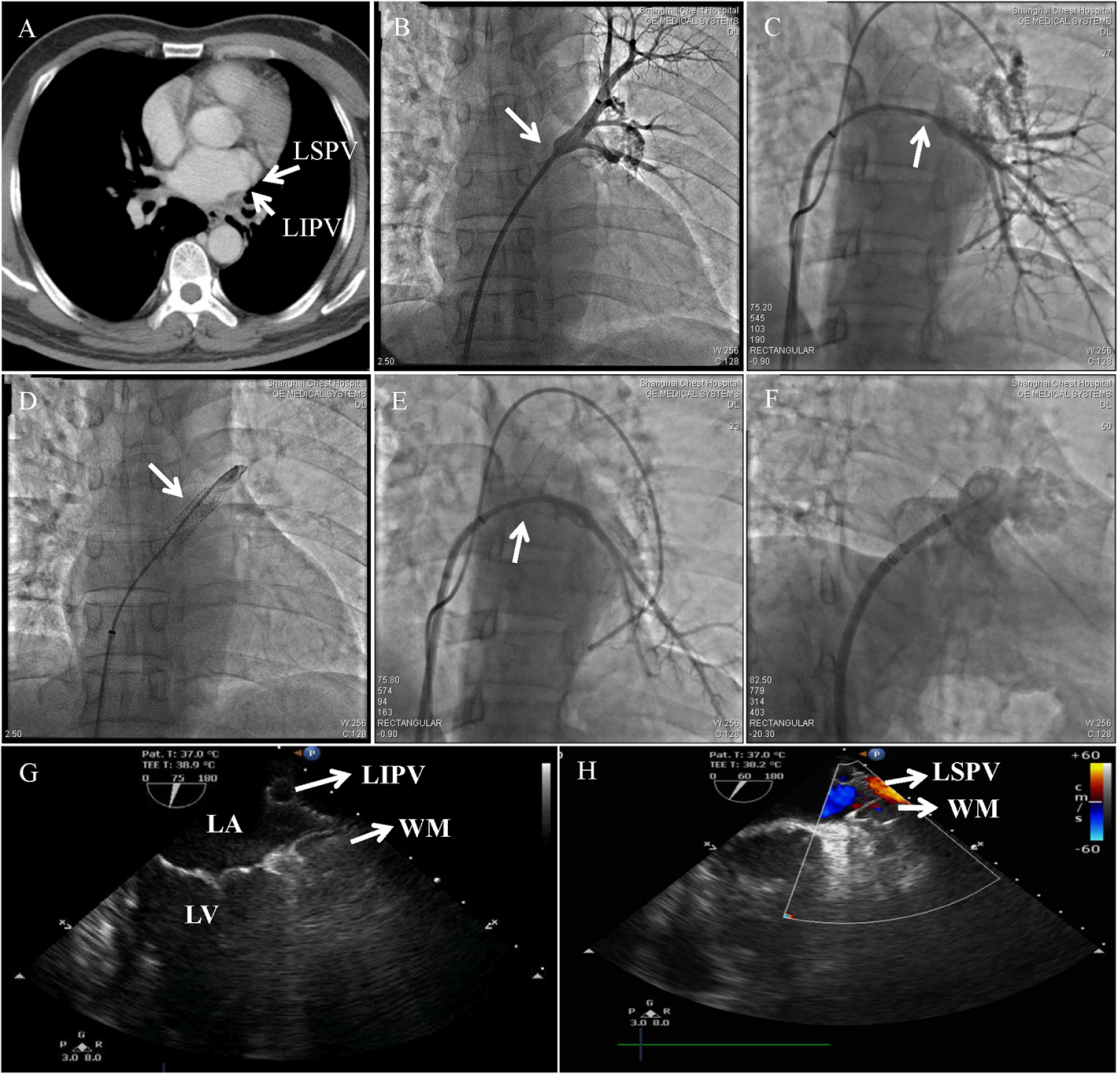
**a** Subtotal stenosis of the left PV in CT scan. **b**, **c** PV angiography shows severe stenosis of the left PV (arrow) and plexiform vein collaterals before PV Stenting. **d**, **e** PV angiography shows patent stents with no residual stenosis (arrow) after PV Stenting. **f** Left atrial appendage angiography. **g**, **h** Partially deployed Watchman device

A 67-year-old man with diabetes, hypertension and a history of stroke underwent radiofrequency pulmonary vein (PV) isolation for persistent AF 1 year ago. Six months later he developed recurrent respiratory infection and massive hemoptysis. Electrocardiogram showed recurrence of AF. Warfarin was initially started but discontinued due to hemoptysis. Metoprolol was used for rate control. Computed tomography pulmonary angiography (CTPA) revealed severe left PV stenosis (Fig. 1a). He was admitted in our hospital for further treatment.

The patient was at high risk of AF-related stroke (CHA_2_DS_2_-VASc score = 5) and bleeding (HAS-BLED score = 3). In addition, risk of PV re-stenosis and recurrence of hemoptysis made long-term oral anticoagulation unfavorable in this patient.

We thus attempted to combine percutaneous PVS stenting and LAAO to resolve the problem. For percutaneous PVS stenting, we started with left pulmonary artery wedge angiography and direct selective PV angiography. As a result, severe stenosis at the ostium of left superior and inferior PV were visualized. New vascular plexus distal to the stenosis was highly suspected as the source of hemorrhage (Fig. 1b, c). Step-by-step balloon dilation of the conduit was firstly performed, and then two relatively big bare metal stents (9*25 mm and 8*27 mm; Boston Scientific, Natick, Massachusetts, USA) were successfully deployed in the proximal left superior and inferior PV respectively. PV angiography demonstrated no residual stenosis, rupture or dissection of the lesion (Fig. 1d, e). The transstenotic pressure gradient was decreased from 22 mmHg to 0 mmHg. Pulmonary vein caliber was significantly increased from 2.1 mm to 8.5 mm, and pulmonary artery pressure was decreased from 52/21 mmHg (31 mmHg) to 32/20 mmHg (24 mmHg). For LAAO, a Watchman Double Curve Access System was advanced into left atrium (LA) over the guidewire. Left atrial appendage (LAA) angiography revealed the whole lobe (Fig. 1f), and the ostial diameter was measured to be 23–25 mm by angiography and transesophageal echocardiography (TEE) views. Thereafter, a 30 mm Watchman occluder was deployed under fluoroscopy and TEE guidance (Fig. 1g, h). Once no significant peridevice leak was found and all release criteria were confirmed, the device was fully released. Heparin (80 IU/kg) was used during the procedure and activated clotting time was maintained as 250 s - 350 s.

**Fig. 2:**
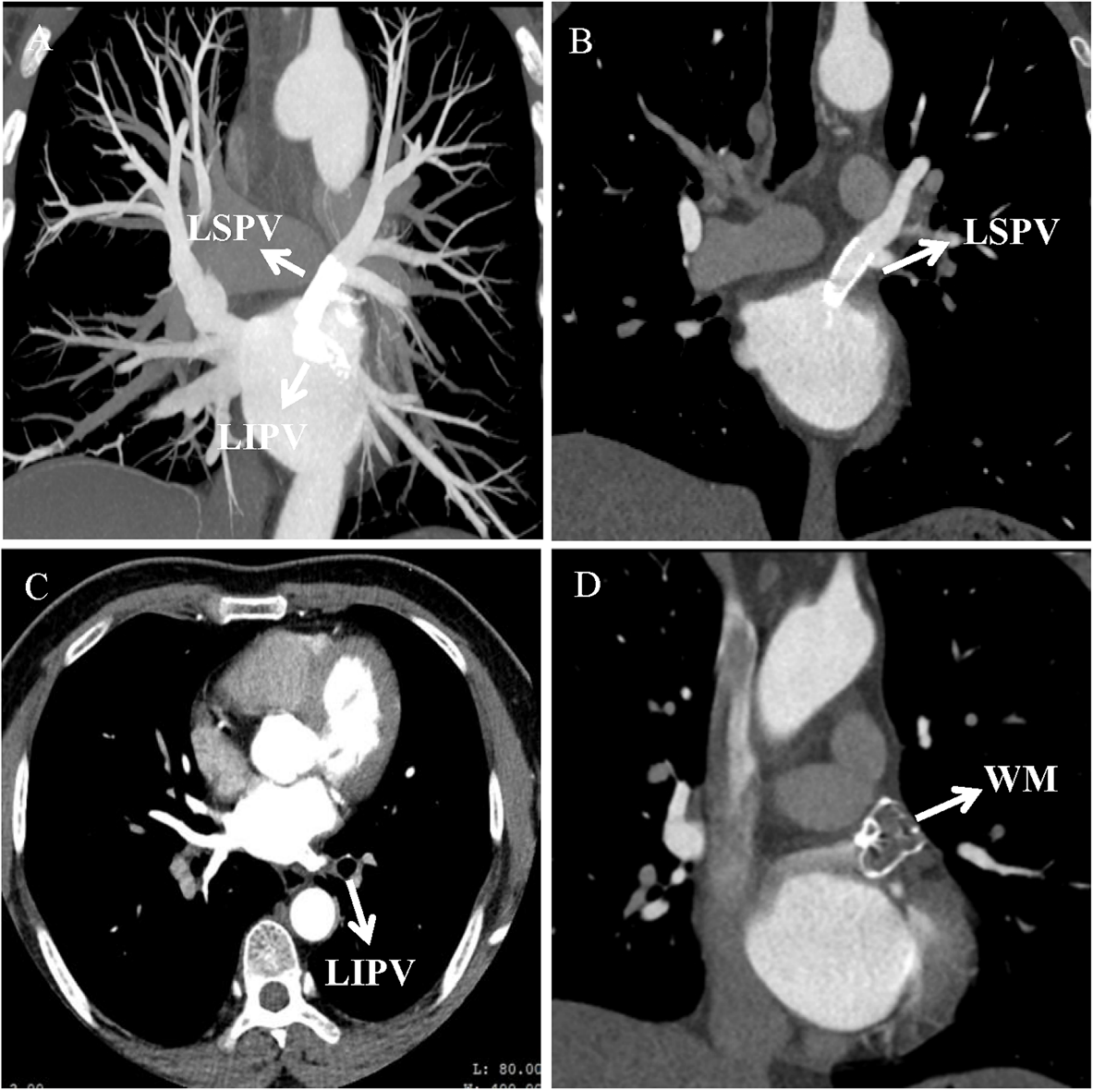
**a** Restored pulmonary blood flow. **b**, **c** No in-stent restenosis. **d** No device-related thrombus in CT scan at 6 months post-procedure

After the procedure, the patient experienced immediate relief of symptoms. Then he received a combination of Aspirin and Clopidogrel for 6 months followed by Aspirin only indefinitely. The patient was followed at 3, 6 and 12 months. He remained asymptomatic without bleeding recurrence. CT scan was performed at 6 months and showed restored pulmonary venous flow (Fig. 2a), no in-stent restenosis (Fig. 2b, c) and no device related thrombosis (DRT) with regard to Watchman (Fig. 2d).

## Discussion and conclusions

PVS after radiofrequency ablation is an uncommon but serious complication [6]. In symptomatic cases, PVS stenting can provide hemodynamic relief of pulmonary circulation [2]. However, like our patient, who had a bleeding complication and relative contraindication to chronic oral anticoagulation with warfarin or NOACs, the potential risk of LAA thrombosis and concomitant stroke are challenging. Re-Do ablations are possible in PVS after stents implantation, but the clinical benefit should be carefully considered and ostial ablation should be avoided [7]. Thus, stroke prophylaxis is of significance in the situation. While this case is performed combining with LAAO, these risks could be substantially reduced [8].

The current case illustrates the safety and feasibility of simultaneous PVS stenting and LAAO in such situation. Our experience of this procedure is that PV stenting should be first successfully performed; left superior PV stent should not be placed too close to the LA body. Sequentially, the Watchman Access Sheath should be advanced slowly to avoid interfering with PV stent.

In conclusion, the strategy of PVS stenting combined with LAAO can solve the dilemma of bleeding and thromboembolism. During the procedure, we deployed the large stent in the stenotic PV followed by dual antiplatelet therapy per the ASAP registry regimen [9]. The patient had good short and mid-term results upon follow up.

## Data Availability

Not applicable.

## Abbreviations

AF: Atrial fibrillation
CT: Computed tomography
CTPA: Computed tomography pulmonary angiography
DAPT: Dual antiplatelet therapy
DRT: Device-related thrombus
LA: Left atrium
LAAO: Left atrial appendage occlusion
LIPV: Left inferior pulmonary vein
LSPV: Left superior pulmonary vein
LV: Left ventricle
PV: Pulmonary vein
PVS: Pulmonary vein stenosis
TEE: Transesophageal echocardiography
WM: Watchman
